# *RTEL1* gene polymorphisms and neuroblastoma risk in Chinese children

**DOI:** 10.1186/s12885-023-11642-3

**Published:** 2023-11-24

**Authors:** Ting Zhang, Chunlei Zhou, Jiejie Guo, Jiamin Chang, Haiyan Wu, Jing He

**Affiliations:** 1 Department of Clinical Laboratory, The Affiliated Wenling Hospital of Wenzhou Medical University, Taizhou, 317500 Zhejiang China; 2 Department of Pathology, Children’s Hospital of Nanjing Medical University, 72 Guangzhou Road, Nanjing, 210008 Jiangsu China; 3 Department of Pediatric Surgery, Guangzhou Institute of Pediatrics, Guangdong Provincial Key Laboratory of Research in Structural Birth Defect Disease, Guangzhou Women and Children’s Medical Center, Guangzhou Medical University, Guangdong Provincial Clinical Research Center for Child Health, 9 Jinsui Road, Guangzhou, 510623 Guangdong China

**Keywords:** *RTEL1*, Polymorphism, Neuroblastoma, Susceptibility

## Abstract

**Background:**

Neuroblastoma, a neuroendocrine tumor originating from the sympathetic ganglia, is one of the most common malignancies in childhood. *RTEL1* is critical in many fundamental cellular processes, such as DNA replication, DNA damage repair, genomic integrity, and telomere stability. Single nucleotide polymorphisms (SNPs) in the *RTEL1* gene have been reported to confer susceptibility to multiple cancers, but their contributing roles in neuroblastoma remain unclear.

**Methods:**

We conducted a study on 402 neuroblastoma cases and 473 controls to assess the association between four *RTEL1* SNPs (rs3761124 T>C, rs3848672 T>C, rs3208008 A>C and rs2297441 G>A) and neuroblastoma susceptibility.

**Results:**

Our results show that rs3848672 T>C is significantly associated with an increased risk of neuroblastoma [CC vs. TT/TC: adjusted odds ratio (OR)=1.39, 95% confidence interval (CI)=1.02-1.90, *P*=0.038]. The stratified analysis further indicated that boy carriers of the rs3848672 CC genotype had a higher risk of neuroblastoma, and all carriers had an increased risk of developing neuroblastoma of mediastinum origin. Moreover, the rs2297441 AA genotype increased neuroblastoma risk in girls and predisposed children to neuroblastoma arising from retroperitoneal.

**Conclusion:**

Our study indicated that the rs3848672 CC and rs2297441 AA genotypes of the *RTEL1* gene are significantly associated with an increased risk of neuroblastoma in Chinese children in a gender- and site-specific manner.

**Supplementary Information:**

The online version contains supplementary material available at 10.1186/s12885-023-11642-3.

## Introduction

Neuroblastoma (NB) evolves from primitive neural crest cells, mostly stemming from the sympathetic nervous system chain and adrenal medulla [1]. NB occurs in ~150-200 children annually in Japan, and the incidence rate is 7.7 per million in China [2]. NB is not only highly heterogeneous but also has a variety of clinical symptoms [3, 4]. The International Neuroblastoma Risk Group (INRG) classifies NB into three risk groups (low-risk, intermediate-risk, or high-risk) based on age (month), tumor grade, histological category, 11q distortion, differentiation, MYCN and ploidy [5, 6]. Currently, the overall survival of NB is about 81% due to the great improvements in patient stratification and treatment regimens [7]. The five-year survival rates of low- and intermediate-risk neuroblastoma patients are as high as 80-90%, depending on the geographic area. Thanks to the emergence of stem cell transplantation, anti-GD2 based immunotherapy, and the differentiating agent isotretinoin, even in the high-risk group, the five-year survival rate of high-risk patients is enhanced by up to 50% [8, 9]. However, it is noteworthy that the majority of patients develop resistance to treatment, eventually experiencing recurrence, and NB survivors are often at risk of developing secondary neoplasms [10–13]. Therefore, there is an urgent need to understand the biological characteristics and pathogenesis of NB better and develop more efficacious therapies.

In recent decades, it has been reported that children exposed to some environmental factors are prone to the development of neuroblastoma, such as the mother's medication, living conditions, children's infection, pregnancy, and pregnancy exposure; however, the causal relationship between the two has not been confirmed [14, 15]. The etiology of neuroblastoma is still largely unknown. Many types of disease-causing genetic alterations are identified through whole genome sequencing and genome-wide association studies (GWASs), including single nucleotide polymorphisms (SNPs), mutations, deletions, amplification, and rearrangements [16, 17]. *MYCN* [18], *ALK* [14], and *PHOX2B* [19] gene mutations have been shown to increase the risk of neuroblastoma. Meanwhile, SNPs in a number of genes are associated with susceptibility to sporadic neuroblastoma, including *TP53* [20], *CASC15* [21, 22], *LIN28B* [23], *TERT* [24], *ATRX* [25], *LMO1* [26], *NBPF* [27], *BARD1* [28] genes and mitochondrial *ND4* gene [29]. Although an increasing number of genetic variants have been identified, the more unknown causal genetic variants still needs to be explored for neuroblastoma.

Regulation of telomere elongation helicase 1 (RTEL1), containing Fe-S clusters, is an essential DNA helicase with 5’-3’ helicase activity. It could disassemble various DNA protein secondary structures to promote DNA repair, telomere maintenance and set telomere length in mice [30]. *Rtel (-/-)* mouse embryonic stem cells exhibit significant telomere deletion and chromosome breakage or fusion during differentiation [31]. Recently, GWAS showed that *RTEL1* gene variations seem to be closely related to various diseases, such as glioma [32], lung cancer [33], astrocytomatal cancer [34], coronary heart [35], interstitial pneumonia [36], and ulcerative colitis [37]. However, no publication has reported a linkage between *RTEL1* gene polymorphisms and the risk of neuroblastoma. To determine the association between neuroblastoma susceptibility and *RTEL1* gene SNPs, we conducted this case-control study in Chinese children.

## Materials and methods

### Study subject

In the present study, we applied structured questionnaires to collect epidemiological data. A total of 402 children with neuroblastoma and 473 healthy controls without cancer history were collected from the Children’s Hospital of Nanjing Medical University during the same period (Table S1) [38, 39]. Children with neuroblastoma who meet the eligibility criteria need to be diagnosed with the confirmation by pathological biopsy. Cases and healthy children were matched by age, gender, and ethnicity. All participants’ parents or guardians signed an informed consent form. The research scheme was approved by the Institutional Review Committee of Children’s Hospital of Nanjing Medical University. The authors are responsible for all aspects of this study.

### Polymorphism selection and genotyping

We identified four potential functions of SNPs (rs3761124 T>C, rs3848672 T>C, rs3208008 A>C, rs2297441 G>A) in the *RTEL1* gene by combining the NCBI dbSNP database and SNPinfo software [40–42]. All selected SNPs were located in exons, introns, splice sites, 5’ untranslated regions (UTRs), or 3’ UTRs of the *RTEL1* gene. They are in low linkage disequilibrium (LD) with each other (R^2^<0.8). LD is referred to as the non-random association of alleles at nearby loci. Variants in high LD (R^2^≥0.8) are highly correlated and can be a proxy of each other being associated with the same phenotypes, such as cancer susceptibility [43]. We chose SNPs in low LD to ensure the identification of maximal disease susceptibility loci with a limited number of SNPs. The minor allele frequencies of these SNPs in Chinese Han subjects are >5%. The rs3761124 T>C is located upstream of the *RTEL1* gene, the rs3208008 A>C and rs3848672 T>C in exons, and the rs2297441 G>A is in the 3’ UTR of the *RTEL1* gene. These polymorphisms may affect *RTEL1* gene expression. Genomic DNA was isolated from the peripheral blood of participants using a TIANamp Blood DNA kit. Then, we genotyped the purified DNA samples of the neuroblastoma group and control group using standard TaqMan real-time PCR. Finally, to ensure reliability, accuracy, and repeatability, we randomly chose 10% of the completed research samples for repeated experiments. The repetition rate of all samples was 100%.

### Statistical analysis

The chi-squared test of goodness of fit was used to determine the Hardy-Weinberg equilibrium (HWE) among the control groups. Bilateral χ^2^ tests were used to assess the differences in the distribution of genotype frequency and other characteristics between the patient and control groups. We applied multiple logistic regression to determine the association between the risk of neuroblastoma and the *RTEL1* gene polymorphisms, with 95% confidence intervals (CIs) and odds ratios (ORs) adjusted by age and sex. The SNP genotype that increases the risk of neuroblastoma (OR>1) was defined as a risk genotype. In addition, we divided the patients into different subgroups based on age, sex, tumor origin, and clinical staging for further stratified analysis. We set 0.05 as the threshold of statistically significant difference for all tests, and all analyses were two-sided. All statistical analyses were calculated using SAS software version 9.4.

## Results

### *RTEL1* gene polymorphisms and neuroblastoma susceptibility

The association between the genotype frequencies of the four *RTEL1* gene polymorphisms (rs3761124 T>C, rs3848672 T>C, rs3208008 A>C, and rs2297441 G>A) and the susceptibility to neuroblastoma are shown in Table 1. We verified that the distribution of all four gene polymorphisms conformed to HWE (*P*>0.05) in controls. After adjusting for age and gender, we observed that rs3848672 T>C was significantly associated with an increased risk of neuroblastoma in the single-locus analysis (CC vs. TT/TC: adjusted OR=1.39, 95% CI=1.02-1.89, *P*=0.038). However, no significant associations were found for the rs3761124 T>C, rs3208008 A>C, and rs2297441 G>A polymorphisms.

**Table 1:** Association between *RTEL1* gene polymorphisms and neuroblastoma susceptibility in children from Jiangsu province

Genotype	Cases (*N=*402)	Controls (*N=*473)	*P*^a^	Crude OR (95% CI)	*P*	Adjusted OR (95% CI)^b^	*P*^b^
rs3761124 T>C (HWE=0.676)							
TT	201(50.00)	260 (54.97)		1.00		1.00	
TC	169 (42.04)	179 (37.84)		1.22 (0.92-1.62)	0.161	1.22 (0.92-1.62)	0.161
CC	32 (7.96)	34 (7.19)		1.22 (0.73-2.04)	0.455	1.22 (0.73-2.04)	0.456
Additive			0.180	1.16 (0.94-1.43)	0.181	1.16 (0.94-1.43)	0.181
Dominant	201 (50.00)	213 (45.03)	0.142	1.22 (0.94-1.59)	0.143	1.22 (0.94-1.59)	0.143
Recessive	370 (92.04)	439 (92.81)	0.667	1.12 (0.68-1.85)	0.666	1.12 (0.68-1.85)	0.666
rs3848672 T>C (HWE=0.874)							
TT	117 (29.10)	135 (28.54)		1.00		1.00	
TC	175 (43.53)	237 (50.11)		0.85 (0.62-1.17)	0.320	0.85 (0.62-1.17)	0.319
CC	110 (27.36)	101 (21.35)		1.26 (0.87-1.81)	0.222	1.26 (0.87-1.82)	0.219
Additive			0.269	1.11 (0.92-1.33)	0.269	1.11 (0.92-1.33)	0.267
Dominant	285 (70.90)	338 (71.46)	0.855	0.97 (0.73-1.31)	0.855	0.97 (0.73-1.31)	0.854
Recessive	292 (72.64)	372 (78.65)	0.038	**1.39 (1.02-1.89)**	**0.039**	**1.39 (1.02-1.90)**	**0.038**
rs3208008 A>C (HWE=0.755)							
AA	172 (42.79)	220 (46.51)		1.00		1.00	
AC	175 (43.53)	203 (42.92)		1.10 (0.83-1.47)	0.500	1.10 (0.83-1.47)	0.501
CC	55 (13.68)	50 (10.57)		1.41 (0.91-2.17)	0.121	1.41 (0.91-2.17)	0.121
Additive			0.138	1.16 (0.95-1.41)	0.138	1.16 (0.95-1.41)	0.138
Dominant	230 (57.21)	253 (53.49)	0.269	1.16 (0.89-1.52)	0.270	1.16 (0.89-1.52)	0.270
Recessive	347 (86.32)	423 (89.43)	0.158	1.34 (0.89-2.02)	0.159	1.34 (0.89-2.02)	0.159
rs2297441 G>A (HWE=0.936)							
GG	177 (44.03)	225 (47.57)		1.00		1.00	
GA	175 (43.53)	203 (42.92)		1.10 (0.83-1.45)	0.525	1.10 (0.83-1.45)	0.525
AA	50 (12.44)	45 (9.51)		1.41 (0.90-2.21)	0.131	1.41 (0.90-2.21)	0.131
Additive			0.153	1.16 (0.95-1.41)	0.154	1.16 (0.95-1.41)	0.153
Dominant	225 (55.97)	248 (52.43)	0.295	1.15 (0.88-1.51)	0.295	1.15 (0.88-1.51)	0.295
Recessive	352 (87.56)	428 (90.49)	0.166	1.35 (0.88-2.07)	0.167	1.35 (0.88-2.07)	0.167
Combined effect of risk genotypes^c^							
0-1	160 (39.80)	218 (46.09)		1.00		1.00	
2-4	242 (60.20)	255 (53.91)	0.061	1.29 (0.99-1.69)	0.062	1.29 (0.99-1.69)	0.062

^a^χ^2^ test for genotype distributions between neuroblastoma patients and controls

^b^Adjusted for age and gender

^c^Risk genotypes were carriers with rs3761124 TC/CC, rs3848672 CC, rs3208008 AC/CC, rs2297441 GA/AA genotypes

### Stratification analysis

First, we divided the study population into different subgroups by age, sex, tumor origin, and clinical stage. Second, we studied the effect of individual rs3761124 T>C, rs3848672 T>C, rs2297441 G>A polymorphisms and combined risk genotypes on the neuroblastoma susceptibility in different subgroups. As shown in Table 2, we detected that compared to the reference genotype, the rs3848672 CC genotype had enhanced effects on neuroblastoma risk in the following subgroups: male (adjusted OR=1.54, 95% CI=1.003-2.36, *P*=0.049) and tumors originating in the mediastinum (adjusted OR=1.94, 95% CI=1.25-3.01, *P*=0.003). Similarly, we found that the rs2297441 AA genotype had a more substantial risk effect in girls (adjusted OR=1.93, 95% CI=1.01-3.67, *P*=0.046) and increased children’s propensity to develop neuroblastoma originating from the retroperitoneal (adjusted OR=1.76, 95% CI=1.05-2.96, *P*=0.033).

**Table 2:** Stratification analysis for association between *RTEL1* gene genotypes and neuroblastoma susceptibility

Variables	rs3848672 (case/control)		AOR(95% CI)^a^	*P*^a^	rs2297441 (case/control)		AOR(95% CI)^a^	*P*^a^	Risk genotypes (case/control)		AOR(95% CI)^a^	*P*^a^
	TT/TC	CC			GG/GA	AA			0-1	2-4		
Age, month												
≤18	98/106	41/33	1.34 (0.79-2.29)	0.279	122/125	17/14	1.25 (0.59-2.64)	0.567	55/64	84/75	1.30 (0.81-2.10)	0.275
>18	194/266	69/69	1.39 (0.95-2.04)	0.091	230/303	33/31	1.40 (0.84-2.36)	0.201	105/154	158/180	1.29 (0.93-1.79)	0.130
Gender												
Female	142/176	49/49	1.24 (0.79-1.95)	0.355	165/208	26/17	**1.93 (1.01-3.67)**	**0.046**	77/105	114/120	1.30 (0.88-1.91)	0.194
Male	150/196	61/52	**1.54 (1.003-2.36)**	**0.049**	187/220	24/28	1.01 (0.57-1.80)	0.974	83/113	128/135	1.29 (0.89-1.87)	0.181
Sites of origin												
Adrenal gland	76/372	17/101	0.83 (0.47-1.48)	0.535	82/428	11/45	1.27 (0.63-2.56)	0.503	37/218	56/255	1.28 (0.82-2.02)	0.281
Retroperitoneal	121/372	46/101	1.40 (0.93-2.10)	0.104	141/428	26/45	**1.76 (1.05-2.96)**	**0.033**	63/218	104/255	1.41 (0.99-2.03)	0.060
Mediastinum	79/372	41/101	**1.94 (1.25-3.01)**	**0.003**	111/428	9/45	0.77 (0.36-1.62)	0.488	49/218	71/255	1.24 (0.83-1.86)	0.302
Others	13/372	5/101	1.39 (0.49-4.01)	0.538	15/428	3/45	1.94 (0.54-6.98)	0.312	9/218	9/255	0.87 (0.34-2.22)	0.764
Clinical stage												
I+II+4s	130/372	43/101	1.25 (0.83-1.88)	0.295	156/428	17/45	1.02 (0.56-1.83)	0.959	75/218	98/255	1.11 (0.78-1.58)	0.556
III+IV	121/372	42/101	1.29 (0.85-1.95)	0.233	141/428	22/45	1.48 (0.86-2.56)	0.157	63/218	100/255	1.35 (0.94-1.94)	0.106

^a^Adjusted for age and gender, omitting the corresponding stratify factor

## Discussion

This research investigated the association between four *RTEL1* gene SNPs and neuroblastoma risk by conducting a case–control study with 402 neuroblastoma patients and 473 healthy controls. Here, we found that rs3848672 T>C could increase the risk of neuroblastoma, especially among boys and subjects with mediastinum-origin tumors. Meanwhile, the rs2297441 G>A polymorphism conferred increased risk in girls and subjects with neuroblastoma of retroperitoneal origin. To the best of our knowledge, the association between *RTEL1* gene polymorphisms and the risk of neuroblastoma in Chinese children has not been reported before.

The telomere structure comprises TTAGGG repeat sequences and their related factors or protective proteins, which are necessary for maintaining genomic stability and human linear chromosome integration [44]. In normal human somatic cells, telomeres are shortened due to cell replication or aging. In contrast, cancer cells proliferate infinitely without losing telomeres. RTEL1 plays an essential role in the process of DNA unwinding. Its encoding gene is located at 20q13.3 and contains 40 exons. *RTEL1* can decompose different kinds of DNA secondary structures and promote DNA repair, replication, and recombination, thus helping to maintain the integrity of telomeres [45]. It has been reported that inactivation of the *RTEL1* gene leads to chromosome breakage, fusion, and telomere loss in mice [46]. The human *RTEL1* gene is a direct homolog of the mouse *RTEL1* gene, and its protein products may have similar effects. When the *RTEL1* gene is deleted in cells, sister chromatid exchange and gene replacement are more likely to occur [47]. *RTEL1* gene-deficient stem cells are prone to local chromosome breaking, and their cell differentiation and proliferation abilities are significantly decreased [48]. In addition, *RTEL1* gene-deficient cells are more sensitive to DNA damage during embryonic development. Therefore, any *RTEL1* SNPs affecting telomere length after birth may be able to exacerbate or delay genetic susceptibility to relevant diseases.

Several GWAS and candidate gene studies have identified that *RTEL1* variants are associated with cancer genetic susceptibility, including lung cancer, breast cancer, gastric cancer, colorectal cancer, and esophageal cancer [49]. A meta-analysis showed a correlation between the G allele of *RTEL1* rs6010620 and an increased risk of glioma, including 1878 cases and 3670 controls [50]. In addition, a mouse model study showed that the *RTEL1* gene could regulate the Wnt/β-Catenin signaling to support cell growth, suggesting that the *RTEL1* may be considered a carcinogenic gene [48]. The above results collectively indicate that the *RTEL1* gene is a cancer-promoting factor and may also be an anti-cancer target. Among the four investigated SNPs (rs3761124 T>C, rs3848672 T>C, rs3208008 A>C, and rs2297441 G>A) in the present study, Egan et al. reported that the rs3208008 was associated with an increased risk of glioma in a US study population [51]. The other three SNPs have not been reported to be related to disease. In this study, we concluded that rs3848672 T>C and rs2297441 G>A significantly increased the risk of neuroblastoma in Chinese children.

Consistent with the vital role of *RTEL1* in malignancies, we report for the first time the association between *RTEL1* polymorphisms and the risk of neuroblastoma. It is assumed that the *RTEL1* polymorphism may change the binding affinity of transcription factors, leading to telomere elongation dysfunction, thus affecting the survival of glioma cells [52]. Therefore, there may be the same pathogenesis of neuroblastoma. Our results showed that rs3848672 T>C and rs2297441 G>A polymorphisms could contribute to an increased neuroblastoma risk. However, the other two SNPs, rs3761124 T>C, and rs3208008 A>C, were not associated with the risk of neuroblastoma. Several limitations should be mentioned in this study. First, the sample size of this study may not be large enough to generate a reliable association between genetic variants and disease risk. A larger sample size could improve the statistical power and the credibility of the conclusions. Second, we evaluated only four SNPs in this research. Many other potential functional *RTEL1* polymorphisms should be investigated in the future. Third, the subjects involved in this study were all from the Chinese population, so the conclusions drawn in this study may not apply to other ethnic people. Fourth, this study only evaluated the effects of genetic alterations on the risk of neuroblastoma without considering the impact of environmental factors.

## Conclusion

In conclusion, we provide the first evidence that polymorphisms rs3848672 T>C and rs2297441 G>A in the *RTEL1* gene are associated with the risk of neuroblastoma in the Chinese population. In the future, studies should be conducted with large sample sizes, considering environmental factors, genetic-environmental interactions, and different races.

### Supplementary Information

**Additional file 1: Table S1.** Demographic characteristics of neuroblastoma patients and cancer-free controls from Jiangsu province.

## Data Availability

All the data are available upon request from the correspondence authors (Jing He or Haiyan Wu).

## Abbreviations

NB: neuroblastoma
INRG: International Neuroblastoma Risk Group
GWAS: genome-wide association study
SNP: single nucleotide polymorphism;
RTEL1: regulation of telomere elongation helicase 1
UTR: untranslated region
LD: linkage disequilibrium
HWE: Hardy-Weinberg equilibrium
OR: odds ratio
CI: confidence interval
